# Prescriptions and Dispensing

**Published:** 1907-11-02

**Authors:** 


### Therapeutics.

### PRESCRIPTIONS AND DISPENSING.

### IV.—MEDICINES IN LIQUID FORM.

The last prescription, in which the vehicle is an infusion, raises the question of whether infusions and decoctions should always be freshly prepared according to the official directions, or whether something may be made to represent the required preparation by the use of the so-called " concentrated infusions " and " concentrated decoctions." It is clear that the doctor who dispenses his own medicines has not the time to make fresh infusions for each patient, and he therefore keeps a stock bottle in concentrated form. There are some infusions, such as that of digitalis, which are only efficient to their full extent when they are quite freshly prepared; when it is desired to give these to a patient the doctor should send his prescription to be made up by a chemist who has all the facilities for making the fresh infusion, unless the doctor is in a position to make it freshly for himself. When the prescription is sent to the chemist it should be expressly noted that the infusion is to be absolutely fresh; for although in large establishments fresh supplies of the commoner infusions are made every morning, and of others as required, in small pharmacies it is often the rule to use the concentrated liquids.

There are certain important points that arise when concentrated infusions are used. The principal thing to be borne in mind is that these preparations are not purely aqueous like the fresh infusions, but contain spirit, usually to the extent of about 25 per cent., to act as a preservative, and in many cases this spirit must be taken into consideration not only in regard to the amount of alcohol that the patient may be receiving, but also in connection with the effect of the spirit upon the solubility of other ingredients in the mixture. Another matter to be noted is that the significance of the word " ad " in the prescription may readily be overlooked; a quantity of concentrated infusion equivalent to the total volume of the mixture may be put in instead of the volume required if the prescription were dispensed exactly as written. Thus, in the last example given, if the usual " 1 to 7 " concentrated infusion of orange were employed, the quantity to be taken is as nearly as possible 5 fluid drachms, and not 6, as sometimes erroneously dispensed. The effects of an error of this kind may be but slight; but sometimes, as in the case of important tinctures, such as those of digitalis or nux vomica, the amount of the latter received by the patient in 24 hours may be sufficiently different from that which is desired to make the matter one of serious import.

There is practically no difference between having more of a soluble salt than will dissolve, and having one which is almost insoluble in the vehicle. The following is a common example of the latter case:—

℞ Magnesii carbonatis ... ... ... ʒij.

Magnesii sulphatis ... ... ... ... ℥ss.

Tincturæ rhei ... ... ... ... ℥ss.

Syrupi zingiberis ... ... ... ... ʒvi.

Aquam menthæ piperitæ ... ... ad ℥vi.

The magnesium sulphate is soluble, the carbonate insoluble; the latter must be rubbed down in a mortar with the syrup of ginger, and some of the peppermint water, then transferred to the bottle, and the mortar rinsed with further small quantities of the water; the magnesium sulphate is dissolved as already described, and the tincture of rhubarb added nearly at the end, the measure used for the latter being rinsed with the last small quantity of peppermint water; a " shake the bottle " label must be used.

There are, however, two groups of conditions that are not quite so simply dealt with; the first is where the insoluble ingredient is so heavy that it sinks too rapidly for the patient to be able to get the proper proportion in a dose; the second is where an insoluble substance is formed by reaction between two soluble or liquid ingredients of the prescription, in which case it is very apt to be formed in clots which cannot be evenly diffused by shaking; in both these conditions special means must be adopted in order that the patient may receive in each dose the exact proportions of each remedy in the prescription.

The methods to be adopted in dealing respectively with heavy insoluble solids, and with solids precipitated, in the form of clots in the process of dispensing, are not identical, though similar in some respects. The following prescriptions present instances of the two points under discussion:—

℞ Bismuthi carbonatis ... ... ... ʒiij.

Tincturæ cardamomorum compositæ ... ℥ss.

Acidi hydrocyanici diluti ... ... ... ʒss.

Aquam. chloroformi ... ... ad ℥vi.

If this is dispensed just as written it will be found that the bismuth salt settles so quickly after shaking up that an ounce of the mixture poured out into a glass will contain considerably less than the 30 grains that ought to be in it; and, even if the dose is taken directly it is poured out, the quantity of the carbonate will be further reduced by a portion remaining in the glass.

℞ Quininæ hydrochloridi ... ... ... gr. vi.

Sodii salicylatis ... ... ... ... ʒij.

Ammonii chloridi ... ... ... ... ʒj

Tincturæ gelsemii ... ... ... ... ʒiss.

Aquam ... ... ... ... ... ad ℥vj.

Double decomposition occurs between the quinine hydrochloride and the sodium salicylate, quinine salicylate being precipitated in the form of a bulky flocculent precipitate.

℞ Tincturæ tolutanæ ... ... ... ... ʒij.

Vini ipecacuanhæ ... ... ... ... ʒij.

Syrupi scillæ ... ... ... ... ℥ss.

Aquam cinnamomi ... ... ... ad ℥iv.

The precipitation which occurs here is not due to chemical action, but to the fact that the tolu balsam which is in solution in the tincture is no longer soluble when the spirit of the latter is diluted with the aqueous medium, and a most unpresentable mixture results, in which much of the tolu clings to the sides of the bottle.

(*To be continued.*)

